# The hidden curves of risk: a nonlinear model of cumulative risk and school bullying victimization among adolescents with autism spectrum disorder

**DOI:** 10.1186/s13034-023-00694-9

**Published:** 2024-01-28

**Authors:** Jin-liang Ding, Ning Lv, Yu-fang Wu, I-Hua Chen, Wen-Jing Yan

**Affiliations:** 1 School of Humanities and Teacher Education, Wuyi University, Wuyishan, 354300 China; 2 National Taipei University of Technology, 222 Mount Wuyi No. 2 Middle School, Wuyishan, 354300 China; 3 School of Psychology, Fujian Normal University, Fuzhou, 350117 China; 4 Chinese Academy of Education Big Data, Qufu Normal University, Qufu, 273165 China; 5 School of Mental Health, Wenzhou Medical University, Wenzhou, 325035 China; 6 Zhejiang Provincial Clinical Research Centre for Mental Illness, Affiliated Kangning Hospital, Wenzhou Medical University, Wenzhou, 325035 China

**Keywords:** School bullying victimization, Autism spectrum disorder, Cumulative risk, Internalizing problem

## Abstract

**Background:**

School bullying victimization (SBV) occurs more frequently in students with autism spectrum disorder (ASD) in general education than in special classes, and there is a cumulative risk effect on SBV exposure among young people with ASD reported by their parents and teachers. However, SBV is a personal experience, the predictive patterns of cumulative risk on SBV reported by themselves and its psychological mechanism remain unclear. This study aims to explore the relationship between cumulative risk and SBV based on self-report, and to test whether internalizing problems mediates this relationship among adolescents with ASD placed in regular classes.

**Methods:**

This study used data from the Taiwan Special Needs Education Longitudinal Study (SNELS) in 2011. The analysis included 508 adolescents with ASD who were in regular classes across Taiwan. The primary variables under study were the quality of friendship interactions, teacher-student relationship, school connection, perceived stigma, the impact caused by the disabilities, internalizing problem, and whether the participants had experienced SBV over the past semester, while control variables were adaptability and social-emotional skills. Established risk factors were summed to form a cumulative risk score.

**Results:**

The cumulative risk was positively associated with SBV. The relationship was characterized by the nonlinear pattern of the quadratic function (negative acceleration model) between cumulative risk and SBV. Internalizing problem played a partial mediating role in the effect of cumulative risk on SBV.

**Conclusions:**

Intervention measures to reduce SBV should include the strategies to reduce the number of risks to which adolescents with ASD in regular classes are exposed, comprehensive prevention targeting each risk factor is needed specially when the number of risks is one or two, and more attention needs to be given to their internalizing problem in various ways.

## Introduction

School Bullying Victimization (SBV) is characterized by recurring aggressive actions—be they physical, verbal, or interpersonal—that take place within a power-differentiated relationship [1]. SBV is particularly prevalent among adolescents with special educational needs [2], especially those with Autism Spectrum Disorder (ASD) [3]. The distinctive vulnerabilities of individuals with ASD—including communication deficits, atypical interests, stereotyped behaviors, and limited friendships—contribute to their increased susceptibility to SBV [3, 4]. These vulnerabilities are exacerbated in mainstream educational settings, where students with ASD encounter SBV more frequently than in specialized environments [5], with a noted peak during middle school years (roughly ages 12–15) [6]. Such experiences are not only immediately distressing but can lead to a cascade of adverse long-term effects, including academic difficulties [7], and mental health challenges such as anxiety, depression [8], loneliness, and even suicidal ideation [9]. Identifying and understanding the array of risk factors that contribute to SBV among adolescents with ASD placed in regular classes is thus a critical step toward developing effective interventions.

According to socio-ecological theory [10], it is vital to identify risk factors associated with increased SBV among adolescents with ASD from different perspectives, covering individual characteristics, school and peers. First, from a developmental perspective, children's characteristics such as age, gender, and severity of ASD symptoms may shape SBV. Researchers have found that boys generally tend to be victimized more than girls [11] and SBV peaks during the age of 12–15 [6]. Others include social vulnerability [12], living in a low-income household [13], severity of Asperger syndrome symptomatology [14] were identified as risk factors for SBV among children with ASD. Notably, high level of perceived stigma of adolescents show many victim characteristics, such as low self-esteem, low self-confidence, and high level of loneliness [15], and do easily attract bullies [25]. Schools, as an important places for students' daily lives, have a significant impact on being bullied [16]. In light of this, research has begun to identify the risk factors at school associated with an increased likelihood of exposure to bullying. For example, Thornberg et al. [17] found that as a negative interpersonal interaction phenomenon in youth, SBV can be predicted by adolescents' negative interpersonal relationships with teachers. Similarly, Goldweber et al. [18] found school connection to be the strongest predictor of being bullied among young people. Because peers play a critical socialization role during childhood and adolescence [19], peer relationships are likely implicated in the consideration of risks and protective processes related to victimization. Reciprocated peer relationships are negatively related to victimization [20], while lack of peer support may prevent adolescents from developing social skills normally, making them more likely to be ostracized by peers and becoming targets of being bullied [21]. As with typically developing children, having close friends protects against SBV [22].

Although these studies largely help us understand SBV in youth from different levels of risks, prior studies have often failed to consider these factors' cumulative effect, potentially leading to an overemphasis on isolated risks [23]. Moreover, risk factors in different fields are often synergistic, and individuals are often faced with risk factors in one filed as well as in another [24]. Although single risks such as peer relationships, school belonging, and perceived stigma have been identified [18, 20, 25], their synergistic effect remains under-examined. In the context of ASD, it is hypothesized that as the number of risk factors to which an individual with ASD is exposed increases, so will their vulnerability to becoming the victim of bullying. It is critical to consider multiple risks simultaneously and examine how they interact to influence their likelihood of experiencing SBV.

Existing literature has shown that the cumulative risk model is the most widely used method for modeling multiple risks [24]. Prior research have found that cumulative risk models could show different functional forms: linear and nonlinear models [26, 27]. A linear model shows that the steady increase in risk is proportional to the outcome [28], suggesting that comprehensive prevention effort is essential. A nonlinear model can be further divided into ''positive acceleration'' and ''negative acceleration'' based on regression coefficients [27]. The positive acceleration model describe a quadratic relationship where there is a disproportionate increase in the SBV mean score beyond a certain threshold as the cumulative risk score increases, making intervention challenging [29]. The negative acceleration model assumes that the impact of new risk factors on individual decreases with the increase of cumulative risk, implying interventions for individuals with a medium number of risk factors is more effective [29]. Therefore, exploring different functional forms between cumulative risk and SBV of adolescents with ASD were important because different functional forms can mean different intervention practices for SBV.

To our knowledge, existing literature has yet to explores vulnerability to bullying from a cumulative risk perspective among young people with ASD except Hebron's work [14]. However, the findings of Hebron's work relied solely on parent or teacher reports of SBV, which may not fully align with the self-reported experiences of adolescents with ASD—a discrepancy that could overlook instances occurring beyond their direct observation [30]. Additionally, cultural differences might also influence the SBV and maladjustment relationship [31], suggesting that Hebron's findings from Western countries may not universally apply, particularly in lower SBV incidence regions like China [32]. Thus, the first aim of this study is to elucidate the cumulative risk effect on SBV based on self-reports from adolescents with ASD in China and to map out the constellation of risks they face in mainstream educational settings.

The same level of cumulative risk does not necessarily result in identical adverse outcomes for everyone [33]. It is thus crucial to identify potential factors that may indirectly link cumulative risk to SBV, such as internalizing problems. Internalizing problems often develop silently, manifesting as anxiety, depression, and loneliness, potentially disrupting typical developmental trajectories [34]. They not only compromise adolescents' well-being but also heighten the risk for peer victimization [35]. Children and adolescents who struggle internally may exhibit behaviors or emit signals that bullies perceive as vulnerabilities, thus becoming easier targets [36]. The burgeoning body of research supports the notion that internalizing problems both contribute to and exacerbate the severity of SBV [37–40].

Moreover, the functional form between cumulative risk and depression shows a positive acceleration model [28, 41]. Such risks not only predict current problematic behavior but also increase future depression [27, 42]. One study has confirmed the relationship between cumulative risks and internalizing problems [43]. These dynamics underscore the importance of understanding the mechanisms through which cumulative risk translates into SBV, particularly in the context of ASD where internalizing problems may be more prevalent due to the inherent social communication challenges and environmental stressors faced by these individuals [44]. There is a significant gap in research exploring how these risks, when aggregated, influence the trajectory and intensity of internalizing problems, and in turn, the occurrence of SBV in populations with ASD. Therefore, the second aim of the current study is to explore the relationship between cumulative risk and SBV by examining the potential mediation of Internalizing problem.

Based on the abovementioned review, we put forth two specific hypotheses as follows:

Hypothesis 1: There is a cumulative risk effect on SBV exposure among adolescent with ASD.

Hypothesis 2: Internalizing problem played a partial mediating role in the effect of cumulative risk on SBV.

## Methods

### Data source

The study employs a secondary analysis of cross-sectional data from the Taiwan Special Needs Education Longitudinal Study (SNELS), initiated by Wang in 2007 and supported by Taiwan's National Science Council [45]. The 2011 SNELS survey was designed in Mandarin Chinese to evaluate the adaptability of students with disabilities in a variety of life and academic contexts. It utilized a nationally representative sample of students with disabilities from across Taiwan, as identified in the Taiwan National Special Education Reporting Network (TNSERN) case list. In SNELS, the sampling was conducted on an individual student basis rather than by schools [45]. This method was chosen to avoid biases in the disability category representation that might result from cluster sampling at the school level. The disability categories for students in SNELS were directly obtained from the TNSERN database, where each student with a disability was diagnosed through school assessments, initial psychiatric evaluation, and a board committee's final diagnosis [46]. The SNELS manual indicates that variables such as the type of disability services, socioeconomic status, school location, and gender were not considered in the sampling framework. This was under the premise that a random sampling process would ensure the sample's distribution of these variables would closely reflect the population's characteristics [45].

**Fig. 1:**
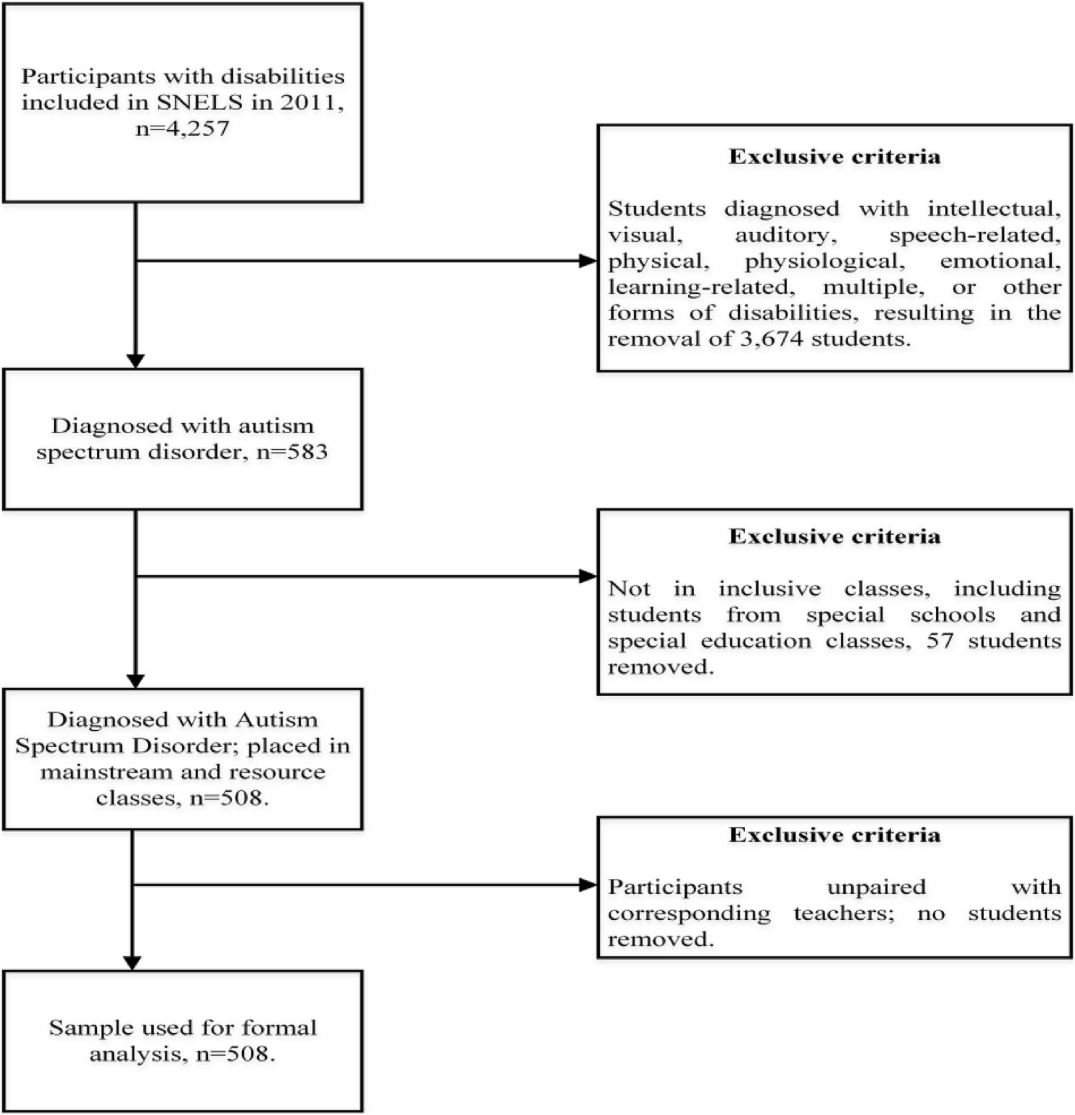
Flowchart elucidating the inclusion and exclusion criteria for the participants

Figure 1 illustrates the selection process used for the SNELS 2011 dataset in this analysis, detailing the criteria for inclusion and exclusion. Initially, the study filtered out students with disabilities other than ASD. It then excluded those not in inclusive education settings. Additionally, cases where student data could not be matched with teacher responses were removed. This step is crucial, as SNELS asserts that teachers, who interact directly with the students, offer a more accurate perspective on assessing students' adaptability. The final sample consisted of 508 adolescents (463 boys) with ASD who were enrolled in regular classes within mainstream schools.

### Measures

In this study, we utilized the SNELS dataset with a focus on the constructs of school bullying victimization (SBV), cumulative risk, and internalizing problems, as outlined in Table 1. We offer a detailed overview of the measures employed for these constructs. To control for potential confounding factors influencing SBV, we included data on the participants' adaptability and social-emotional skills. Recognized as covariates in relation to students' problem behaviors [47, 48], these variables were assessed from the teachers' perspective and served as control variables in our analysis. The reliability of the constructs was determined using ordinal alpha, which is appropriate for the ordinal nature of the item responses, as referenced in [49] and details regarding this are provided later in the methods section. The validity of these constructs is discussed in the results section.

**Table 1:** Questionnaire items and corresponding constructs

Variables	Items
Victimization	*Please select the option that best fits your situation for the following events at school ① Never ② Rarely ③ Sometimes ④ Often* *a. In the school environment, do your peers disregard you?* *b. In the school environment, do your peers insult or ridicule you?* *c. In the school environment, do your peers attempt to extort money from you?* *d. In the school environment, does anyone touch you in a way that causes discomfort?*
The quality of friendship interactions	*In the context of your school, would you describe your interaction with classmates as enjoyable?* *① Extremely enjoyable ② Moderately enjoyable ③Somewhat unenjoyable ④ Not enjoyable at all*
Teacher-student relationship	a. *Do the teachers at your school assist in your learning? ① All do ② Most do ③ Some do ④ Few do* b. *Do you like the teachers at this school? ① Like all ② Like most ③ Like some ④ Don't like any*
School connection	a. *When you encounter difficulties, are there people in the school who help you? ① Many people ② Some people ③A few people ④ Almost no one* b. *Do you feel that you have learned a lot at school? ① A lot ② Some ③ Very little ④ Almost nothing* c. *Do you consider this school to be a safe place? ① Very safe ② Somewhat safe ③ Not very safe ④ Not safe at all* d. *Do you enjoy going to school? ① Very much ② Somewhat ③ Not very much ④ Not at all*
Perceived stigma	a. *At school, do your peers perceive you as different? ① No ② Yes* b. *Do you perceive the teachers at your school to treat you the same as other students? ① Completely the same ② Mostly the same ③ Somewhat different④ Significantly different*
The impact caused by the disabilities	*What is the extent of the impact (① No impact ② Minor impact ③ Moderate impact ④ Significant impact) that this student's disability has on the following aspects?* a. Academic learning b. Self-confidence or self-esteem c. Interpersonal relationships d. Behavior e. Self-care f. Physical health g. Leisure and entertainment
Internalizing problems	a. *How have you been feeling recently? ① Very good ② Okay ③ Not so good ④ Very bad* *Please select the response that best reflects your situation this semester: ① Never ② Rarely ③ Sometimes ④ Often* b. *Have you often been having trouble sleeping and feeling tired?* c. *Have you often been feeling anxious?* d. *Have you often been finding it hard to concentrate?* e. *Have you often been not wanting to socialize with others?* f. *Have you often felt like yelling, throwing things, arguing, or hitting someone?* g. *Have you often felt lonely and helpless?*
Adaptability	a. *The student will make appropriate choices and decisions on their own* b. *When encountering difficulties, the student will find ways to solve them* c. *The student can manage their own time* d. *The student can complete their own responsibilities/tasks*
Social-emotional skills	a. *The student can appropriately express their feelings* b. *The student can manage their emotions or stress* c. *The student understands their strengths and weaknesses in terms of abilities*

### School bullying victimization

SBV assessment in SNELS includes four items evaluating the extent of bullying endured by participants at school, covering relational, verbal, and physical bullying. Scores were tallied on a four-point Likert scale, with higher scores indicating a greater severity of SBV experienced within the school context. The total of these scores served as the SBV indicator. The ordinal alpha for measuring school bullying victimization was 0.62.

### Cumulative risk

Cumulative risk was assessed via five variables, including the quality of friendship interactions, teacher-student relationships, school connection, experiences of stigma, and the impact of disabilities on learning and daily life. Each variable, except for the quality of friendship interactions (which was assessed with a single item), was measured using multiple items on Likert-type scales, where higher scores indicated greater risk. The scores of all items within each construct were summed to represent that construct. Those scores exceeding the 75th percentile among all participants were identified as risks and coded as ''1''; otherwise, they were coded as ''0''. The five risk indices were combined to form the cumulative risk index (see Table 1). The ordinal alpha of the constructs was 0.66 for teacher-student relationship, 0.73 for school connection, 0.68 for perceived stigma and 0.85 for the impact of disabilities.

### Internalizing problems

Internalizing problems were assessed via seven indicators including mood, sleep disturbances, anxiety, concentration difficulties, reluctance to engage with others, uncontrollable behaviors, and feelings of loneliness and helplessness. All indicators, except mood, were rated on a four-point Likert scale from ''never'' (1) to ''often'' (4), while mood was rated from ''very good'' (1) to ''very bad'' (4). The sum of these item scores served as the overall internalizing problems score, with higher scores signifying greater severity. The ordinal alpha was 0.86 for internalizing problems.

### Adaptability

As delineated by Martin et al., adaptability encompasses the cognitive, behavioral, and emotional adjustments individuals make in response to new and uncertain situations [50]. This capacity for adaptation reflects a person's comprehensive ability to navigate the fluctuations of everyday life. A primary aim of the Special Needs Education Longitudinal Study (SNELS) was to assess the adaptability of students with disabilities within their daily routines. Consequently, the survey incorporated items to evaluate the participants' competencies in this area [45]. Four items, detailed in Table 1, were included in the SNELS, prompting teachers to rate the extent of each student's decision-making ability, problem-solving resilience, time management, and task organization on a Likert scale ranging from 1 (strongly agree) to 4 (strongly disagree), with higher scores denoting lower adaptability. The adaptability measure yielded an ordinal alpha of 0.84.

### Social-emotional skills

Social-emotional skills, rooted in innate traits and learned from experience, shape how individuals think, feel, and interact, crucial for personal and social development [51]. These skills typically include understanding and managing emotions, solving social problems, and exhibiting positive behaviors [52]. In the SNELS study, teachers assess these skills in students with disabilities using three targeted items (detailed in Table 1), measured on a four-point Likert scale from ''strongly agree'' to ''strongly disagree'', with higher scores indicating lower social-emotional proficiency. The internal consistency for the social-emotional skills, as measured by ordinal alpha, yielded a coefficient of 0.79.

### Data analysis

Data analysis was conducted with SPSS 23.0 and the R package within JAMOVI [53]. First, participant demographics were analyzed. Second, confirmatory factor analysis (CFA) was then administered to evaluate the factorial validity and convergent validity of the majority of the study's variables—namely, teacher-student relationships, school connections, perceived stigma, the impact of disabilities, and internalizing problems. The constructs of victimization and the quality of friendship interactions were exempted from this analysis. This is because the validity testing of a single item, such as the quality of friendship interactions, via CFA lacks meaningful interpretation. Moreover, the victimization measure aligns more with causal indicators (i.e., formerly formative measurement), with items typically being independent events, thus rendering them unsuitable for CFA [44, 53].

After assessing the quality of the measurement, we calculated descriptive statistics and bivariate correlations to explore the associations between various risk factors and SBV. It is important to note that the sampling strategy implemented by SNELS was centered on individual students with disabilities, rather than on a cluster sampling approach at the class or school level [45]. Additionally, the intra-class correlation coefficient (ICC) for SBV was found to be negligible, registering close to zero (indeed, it was calculated at -0.14). This low ICC indicates that the variance between two randomly chosen individuals from any class is nearly as significant as the variance between two individuals selected at random from the entire population. These two factors—the predetermined individual-based sampling strategy by SNELS and the minimal ICC—led us to determine that Hierarchical Linear Modeling (HLM) was not the appropriate method for our data analysis. Consequently, we employed hierarchical regression analysis, controlling for variables such as gender, adaptability, and social-emotional skills, to examine the impact of cumulative risk. We also investigated the potential mediating effect of internalizing problems in the relationship between cumulative risk and SBV.

Moreover, in the assessment of Perceived Stigma and the Impact of Disabilities, there were instances of missing data for several response items. To determine the nature of this missing data, Little's Missing Completely at Random (MCAR) test was employed. The results yielded a chi-square value of 27.44 (*df* = 26) with a p-value of 0.39. This indicates that the data missingness can be considered as completely at random. Given this finding, the Monte Carlo Markov Chain (MCMC) method, available in the PRELIS module of LISREL, was utilized for multiple imputation.

## Results

### Sample characteristics

Table 2 illustrates that a significant majority of the participants' parents have attained educational levels beyond university. However, it should be noted that approximately 10% of the participants' parents have education levels that only reach elementary and junior high school. In addition, a substantial 90% of these parents are employed full-time, reflecting a strong presence in the workforce. Financially, a notable 56.89% of the families earn a monthly income that exceeds 50,000 NTD (New Taiwan Dollars), indicating a middle to upper-middle economic status.

**Table 2:** Demographic characteristics of the participants (*n* = 508)

	*n* (%)
Father's highest level of education	
Elementary and Junior High School	57 (11.22)
Senior High School	162 (31.89)
University	221 (43.50)
Graduate school	68 (13.39)
Mother's highest level of education	
Elementary and Junior High School	48 (9.44)
Senior High School	195 (38.39)
University	242 (47.64)
Graduate school	23 (4.53)
Father's employment status (Fully employed)	479 (94.29)
Mother 's employment status (Fully employed)	474 (93.31)
Family monthly income (TWD)	
Less than 20,000	27 (5.32)
20,000 to 50,000	192 (37.79)
50,000 to 100,000	170 (33.46)
More than 100,000	119 (23.43)
Sex	
Male	463 (91.1)
Female	45 (8.9)
Grade	
Grade 7	223 (43.9)
Grade 10	166 (32.7)
Grade 12	119 (23.4)
School location	
North region	250 (49.3)
Central region	154 (30.3)
South, east region, and island	104 (20.4)

Demographically, the participant pool is predominantly male, with 91.1% representation. The majority of these participants, 76.6%, are in their first year of junior or senior high school, suggesting a critical period in their educational trajectories. A significant observation is that nearly half of the students diagnosed with Autism Spectrum Disorder (ASD), 49.3%, are enrolled in schools located in Northern Taiwan. This region is distinguished for its superior educational infrastructure, which may play a role in supporting the educational needs of students with ASD.

### Confirmatory factor analysis

**Fig. 2:**
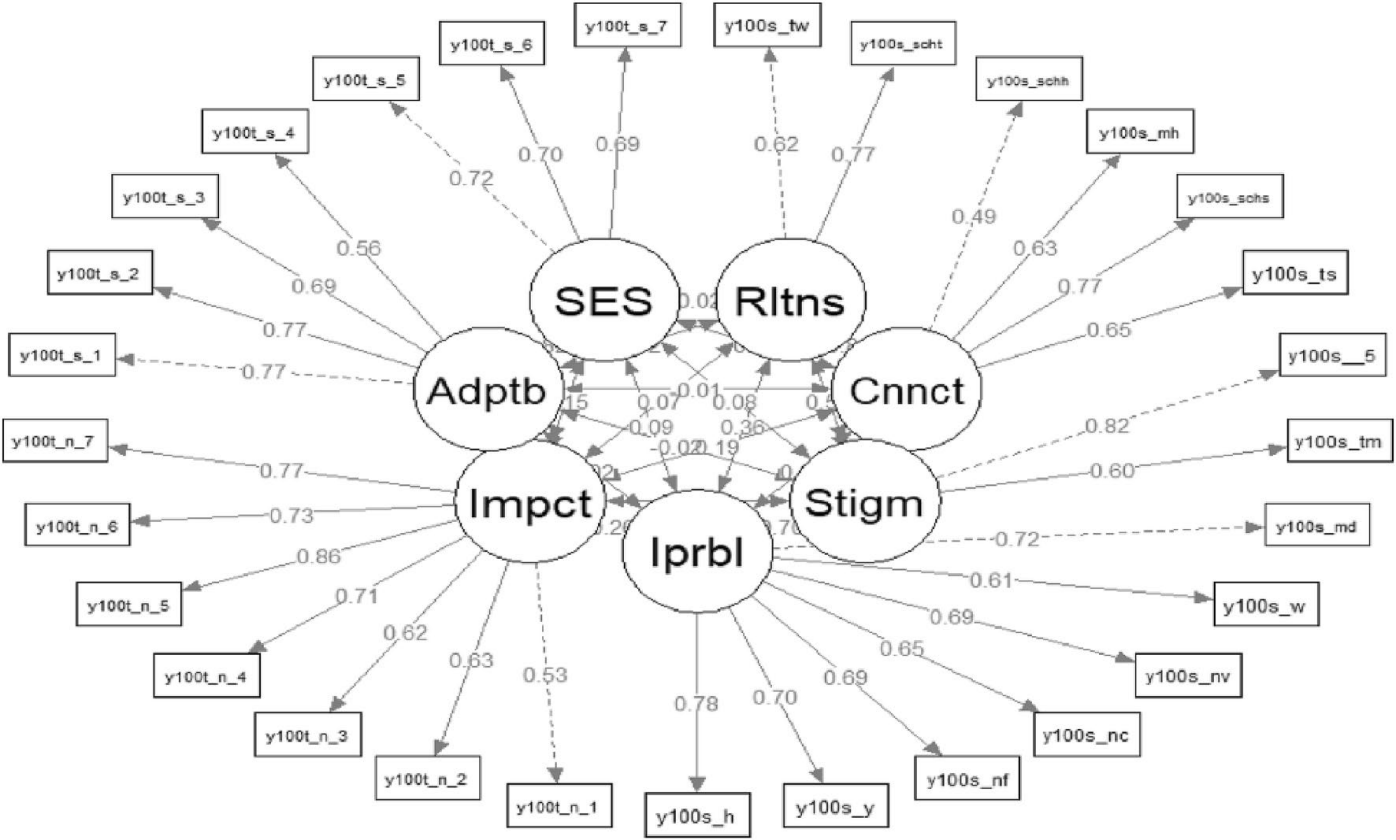
Confirmatory factor analysis results for selected constructs. Note: Indicator titles remain unchanged for consistency with SNELS' released raw data. Rltns: Teacher-student relationship, Cnnct: School connection, Stigm: Perceived stigma, Iprbl: Internalizing problems, Impct: The impact caused by the disabilities, Adptb: Adaptability, SES: Social emotional skills. Note: In the figure, the dashed lines representing factor loadings indicate fixed indicators used to estimate the parameters in confirmatory factor analysis (CFA)

The CFA results indicated that the measurement model possessed satisfactory factorial validity, as evidenced by the acceptable model fit, which did not require the addition of any correlated error terms. Specifically, the chi-square statistic (χ^2^) for the model was 532.82 with 356 degrees of freedom. The Comparative Fit Index (CFI) and the Tucker-Lewis Index (TLI), formerly known as the Non-Normed Fit Index (NNFI), were notably high at 0.983 and 0.980, respectively, indicative of an excellent fit. The Root Mean Square Error of Approximation (RMSEA) was reported as 0.044, with a 95% confidence interval ranging from 0.036 to 0.052, underscoring the precision of the model's fit. Additionally, the Standardized Root Mean Square Residual (SRMR) was 0.063. The factor loadings of the items, most of which were higher than 0.60, are displayed in Fig. 2. Composite Construct Reliability (CCR) was calculated based on the factor loadings shown in Fig. 2, and satisfactory convergent validity was confirmed with CCR values exceeding 0.60 for all constructs: Teacher-Student Relationship at 0.65, School Connection at 0.73, Perceived Stigma at 0.68, Internalizing Problems at 0.87, Impact of Disabilities at 0.87, Adaptability at 0.79, and Social-Emotional Skills at 0.75. These statistical indices collectively suggest a robust and reliable model fit. In summary, the study demonstrates that the data drawn from the Special Needs Education Longitudinal Study (SNELS), used as indicators for the constructs, provides robust support for the measurement validity within the context of the analysis.

### Descriptive statistics and correlations

Table 3 illustrates the extent of cumulative risk encountered by the participants. The findings indicate that no participant in the study exhibited a high level of risk, as none of their scores surpassed the midpoint of the Likert scale across all risk factors. All cumulative risks exhibited a statistically significant positive correlation with SBV, with correlation coefficients (*r* values) ranging from 0.20 to 0.48. The quality of friendship interactions surfaced as the variable with the most significant correlation. Moreover, the five risk factors also displayed significant positive correlations with each other, with one exception: the impact caused by the disabilities. As rated by the teachers, this factor did not exhibit significant correlations with the other cumulative risk factors.

**Table 3:** Descriptive statistics and correlations among risk factors and SBV

	*M* ± *SD*	1	2	3	4	5	6	7	8
1. The quality of friendship interactions	2.05 ± 0.71	–							
2. Teacher-student relationship	4.61 ± 1.41	0.28^**^	–						
3. School connection	7.71 ± 2.08	0.48 ^**^	0.54^**^	–					
4. Perceived stigma	3.91 ± 1.22	0.33^**^	0.30^**^	0.33^**^	–				
5. The impact caused by the disabilities ^a^	15.98 ± 3.92	0.09	0.03	0.11	0.10	–			
6. SBV	6.94 ± 2.08	0.48^**^	0.20^**^	0.33^**^	0.29^**^	0.23^**^	–		
7. Adaptability ^a^	9.43 ± 2.38	− 0.05	0.04	0.05	0.04	0.46^**^	0.03	–	
8. Social-emotional skills ^a^	7.79 ± 1.75	0.05	0.00	0.08^*^	0.05	0.40^**^	0.10^*^	0.61^**^	–

^a^ evaluated by teacher; ^**^p < 0.01, ^*^p < *0.05*

### Hierarchical regression

**Fig. 3:**
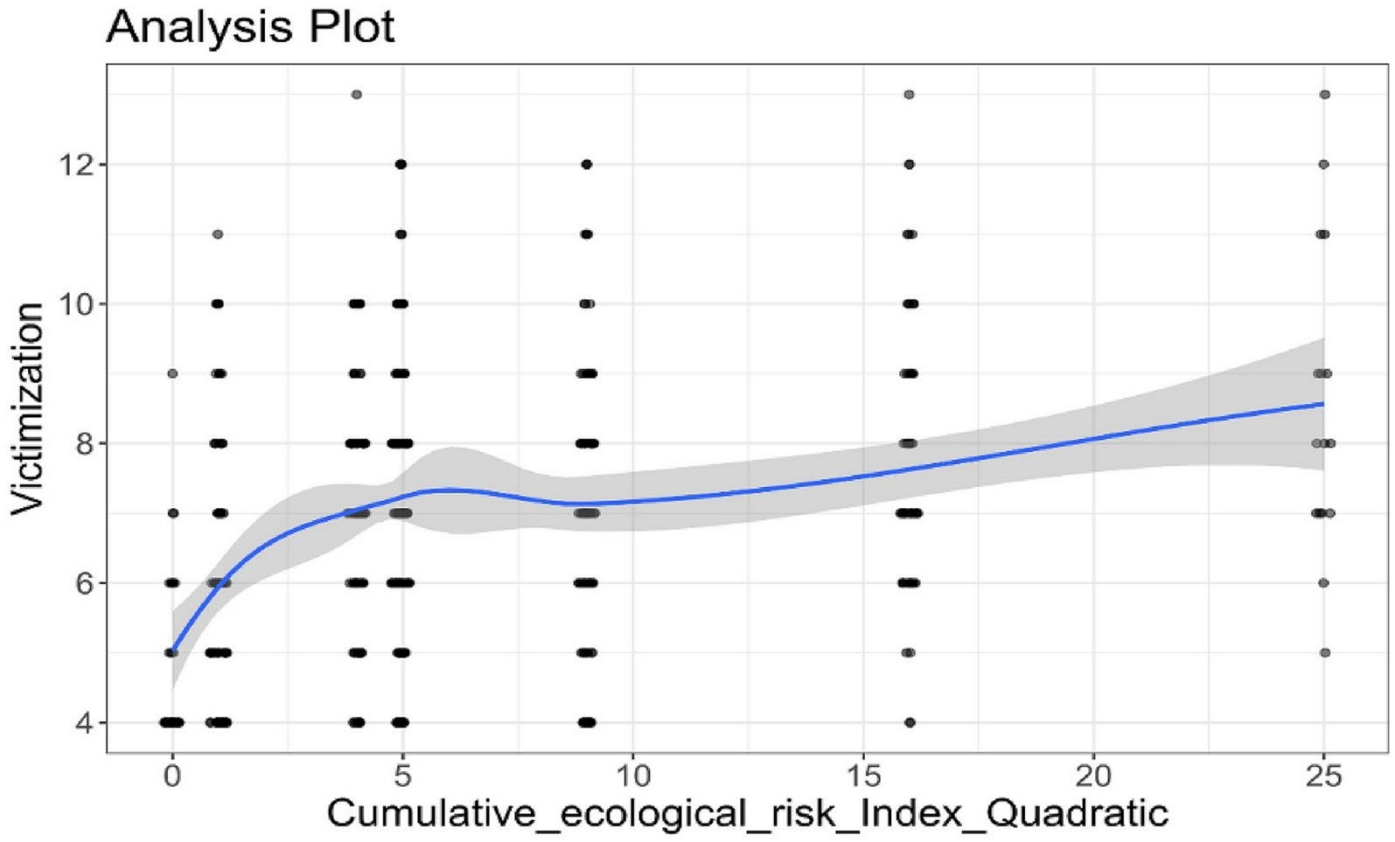
The relationship between cumulative ecological risk index and victimization

In examining the influence of the cumulative risk on SBV, the zero-correlations offered preliminary evidence of significant positive associations among SBV, and cumulative risk, including both linear and quadratic terms (see Table 4). Hierarchical regression results confirmed that, after controlling for gender, adaptability, and social emotional skills, cumulative risk significantly influenced SBV both linearly (*B* = 1.14, SE = 0.24, 95% CI [0.67, 1.60]) and quadratically (*B* = − 0.11, SE = 0.05, 95% CI [− 0.20, − 0.02]) (see Table 5), indicating the relationship was characterized by the nonlinear pattern of the quadratic function (negative acceleration model) between cumulative risk and SBV. Figure 3 also shows that the increase in victimization was not linear across all risk levels. There was a larger change in SBV between 0 and 2 risk levels compared to other risk points.

**Table 4:** The descriptive statistics and Pearson correlations among SBV, cumulative risk, and internalizing problem

	*Mean* ± *SD*	1	2	3	4	5	6
1. SBV	6.94 ± 2.08	–					
2. Cumulative risk	2.27 ± 1.13	0.34^**^	–				
3. Cumulative risk squared	6.42 ± 5.61	0.29^**^	0.95^**^	–			
4. Internalizing problems	15.89 ± 4.40	0.37^**^	0.37^**^	0.35^**^	–		
5. Adaptability	9.43 ± 2.38	0.10^*^	0.10^*^	0.12^*^	0.08^*^	–	
6. Social-emotional skills	7.79 ± 1.75	0.03	0.08^*^	0.10^*^	0.06	0.61^**^	–

^**^p < 0.01, ^*^p < 0.05

**Table 5:** Hierarchical regression on SBV

	Model 1			Model 2			Model 3		
	*B* (SE)	95% CI		*B* (SE)	95% CI		*B* (SE)	95% CI	
		Lower	Upper		Lower	Upper		Lower	Upper
Variables									
Gender (Male)	− 0.43 (0.32)	− 0.51	0.09	− 0.52 (0.31)	− 0.54	0.04	− 0.49 (0.30)	− 0.52	0.05
Adaptability	− 0.12 (0.10)	− 0.17	0.05	− 0.13 (0.09)	− 0.17	0.03	− 0.11 (0.09)	− 0.16	0.04
Social-emotional skills	0.28 (0.10)	0.05	0.26	0.22 (0.09)	0.02	0.22	0.22 (0.09)	0.02	0.22
Cumulative risk				0.62 (0.07)	0.25	0.42	1.14 (0.24)	0.37	0.87
Cumulative risk squared							− 0.11 (0.05)	− 0.55	− 0.04
*R*^2^	0.02			0.13			0.14

### Mediational analysis

After adjusting for demographic variables such as gender, as well as cognitive skills (i.e., adaptability) and social-emotional skills, the findings presented in Table 6 suggest an association where the linear term of cumulative risk is linked with higher levels of internalizing problems (*B* = 1.56, *t* = 3.16, *p* < 0.01). Furthermore, internalizing problems appear to be associated with an increase in SBV (*B* = 0.13, *t* = 6.36, *p* < 0.01). There is also a noted relationship between the linear term of cumulative risk and SBV (*B* = 0.94, *t* = 4.12, *p* < 0.01). The data suggests a potential indirect pathway from cumulative risk to SBV via internalizing problems: The total and direct associations were 0.62 and 0.51, respectively, with a mediating effect of 0.11, SE = 0.07, *p* < 0.001; 95% CI [0.08, 0.35]. This mediating effect accounts for approximately 17.74% of the total association observed.

**Table 6:** Mediation model of internalizing problem

	*B*	SE	*t*	BC 95% CI	
				Lower	Upper
Mediator variable model					
Gender (Male)	− 1.19	0.64	− 1.87	− 2.44	0.06
Adaptability	0.11	0.13	0.81	− 0.15	0.36
Social-emotional skills	− 0.01	0.09	− 0.07	− 0.19	0.18
Cumulative risk	1.56	0.49	3.16	0.59	2.53
Cumulative risk squared	− 0.03	0.10	− 0.26	− 0.22	0.17
Dependent variable model					
Gender (Male)	− 0.35	0.29	− 1.21	− 0.93	0.22
Adaptability	0.11	0.06	1.78	− 0.01	0.22
Social-emotional skills	− 0.05	0.04	− 1.09	− 0.13	0.04
Cumulative risk	0.94	0.23	4.12	0.49	1.39
Cumulative risk squared	− 0.11	0.05	− 2.34	− 0.19	− 0.02
Internalizing problem	0.13	0.02	6.36	0.09	0.17

BC 95% CI = Bias-Corrected 95% Confidence Interval with bootstrap = 5000

## Discussion

The current study underscores the role of cumulative risk as a significant predictor of heightened school bullying victimization (SBV) among adolescents with Autism Spectrum Disorder (ASD) in mainstream classes. Furthermore, it delineates the nonlinear pattern of the quadratic function (negative acceleration model) between cumulative risk and SBV, while also highlighting the direct and indirect influence of cumulative risk on SBV via the mediation of internalizing problems.

Mirroring earlier studies [14], our results demonstrate that a higher cumulative risk correlates with increased SBV among adolescents with ASD. Moreover, we observed a nonlinear pattern (quadratic function) with a negative acceleration between cumulative risk and SBV. Specifically, the rate of SBV exposure escalates substantially with an increasing number of cumulative risks until it reaches a saturation point (four risks in this study), beyond which further increases in risk factors have a diminished ''plateau'' effect. This can be explained from two perspectives. First, when an individual faces risks from at least two domains concurrently, the risk of SBV exposure is severely augmented. Once the cumulative risk reaches a certain threshold, the impact of additional risk factors on bullying saturates. Second, following the study by Jones et al. [43], the ''trigger point'' for substantial adjustment difficulties falls between three and four risks. Therefore, SBV increases linearly with the number of cumulative risks at lower risk levels (e.g., two or three risks). Upon reaching the trigger point (four in this study), the rate of SBV escalates sharply, plateaus, and maintains a relatively high level. These findings provide tentative support for the cumulative risk hypothesis regarding the SBV of adolescents with ASD [41, 42] and offer evidence for intervention strategies targeting SBV.

With regard to the mediating effect of internalizing problems, we found that cumulative risk could lead to conditions such as depression, anxiety, and social phobia, which in turn could precede subsequent SBV. Consistent with prior research [28], we determined that cumulative risk is positively associated with internalizing problems. Socio-ecological theory [54] asserts that human development is influenced by multiple ecological subsystems, such as family, school, and peers. Long-term exposure to a poor school climate [6], unhealthy family environment [55], and weak interpersonal relationships [11] can lead to various internalizing problems. These internalizing issues can negatively affect peer relationships, prompt stronger emotional reactions in ambiguous situations, and contribute to increases in SBV [56]. By contrast, individuals exposed to fewer cumulative risks experience lower levels of internalizing problems, which can help in avoiding SBV. These findings underscore the pivotal role of internalizing problems in the pathway from cumulative risk to SBV.

The findings of this study have critical implications for intervention programs targeting adolescents with ASD experiencing SBV. Firstly, increased attention should be paid to external factors, as a growing body of research points to the importance of contextual risk factors [4, 15]. Interventions need to extend beyond individual-level risk factors and address contextual influences that potentially heighten the risk of adolescents being bullied. For instance, recent studies have shown the effectiveness of parent-assisted learning in developing social skills among children with ASD [57], and interventions with peer groups have been found to impact SBV [15]. Secondly, it is essential to consider the number of identified risks to which adolescents with ASD are exposed, and interventions should aim at reducing the overall number of risks in their lives rather than focusing solely on the specific risks present. Finally, efforts should be made to mitigate internalizing problems in adolescents with ASD. Recommendations have been made for children with ASD who experience internalizing problems to seek help from mental health agencies [58]. Psychotherapy approaches for children and their families have been shown to be beneficial in improving relationship skills [57], which are often areas of difficulty for children with ASD.

Despite the significant contributions of this study, several limitations should be addressed in future research. Firstly, as our study relies on cross-sectional data, we were unable to obtain comprehensive longitudinal data, which is crucial for establishing causality. Secondly, our measure of multiple risk, the cumulative risk index, is additive and does not consider potential interactions between risk factors or the differential impact of individual risks on the outcome variable [23]. More nuanced indices of multiple risk exposure could improve the validity of future studies. Furthermore, due to the limitations of secondary data analysis, certain potential confounding variables, such as ethnicity and IQ, were not included as they were not surveyed in SNELS. Despite controlling for students' adaptability and social-emotional skills, the omission of other confounders remains a limitation of this study. Thirdly, our focus on ASD restricts the generalizability of our findings. Subsequent studies should seek to replicate our findings with other populations. Lastly, although the variance explained by each model was statistically significant, it was relatively small. Future research should consider other risk factors that are not included in the current risk indexes for a more comprehensive understanding of adolescents with ASD of bullying victimization.

## Conclusion

This study examined the cumulative risk hypothesis in relation to SBV among adolescents with ASD, and unveiled the nonlinear pattern of the quadratic function (negative acceleration model) between cumulative risk and SBV. Moreover, it established the mediating role of internalizing problems in the relationship between cumulative risk and SBV. The findings suggest that interventions should aim to reduce the overall number of risks, as opposed to focusing solely on specific risks to which adolescents with ASD in mainstream classes are exposed, as this may be a more realistic and effective strategy. Furthermore, there is a clear need to provide training to parents and teachers on effective strategies for intervening in instances of SBV and addressing victims' internalizing problems. As the first study to examine cumulative risk effects on SBV of adolescents with ASD in regular classes based on self-report, our findings require replication. However, it is hoped that this evidence will contribute to prevention of SBV among this highly vulnerable group of adolescents.

## Data Availability

The data that support the findings of this study are available in the Special Needs Education Longitudinal Study (SNELS) database, released from the Survey Research Data Archive of Academia Sinica. The datasets generated and/or analysed during the current study are not publicly available due to the authors are still working on them but are available from the authors upon reasonable request and with permission of the Survey Research Data Archive of Academia Sinica.

## Abbreviations

ASD: Autism spectrum disorder
SNELS: Special needs education longitudinal study
SBV: School bullying victimization
CFA: Confirmatory factor analysis
Rltns: Teacher-student relationship
Cnnct: School connection
Stigm: Perceived stigma
Iprbl: Internalizing problems
Impct: The impact caused by the disabilities
